# Using GPS Tracking to Investigate Outdoor Navigation Patterns in Patients With Alzheimer Disease: Cross-sectional Study

**DOI:** 10.2196/28222

**Published:** 2022-04-21

**Authors:** Vaisakh Puthusseryppady, Sol Morrissey, Min Hane Aung, Gillian Coughlan, Martyn Patel, Michael Hornberger

**Affiliations:** 1 Norwich Medical School University of East Anglia Norwich United Kingdom; 2 Department of Neurobiology and Behavior University of California Irvine Irvine, CA United States; 3 School of Computing Sciences University of East Anglia Norwich United Kingdom; 4 Rotman Research Institute Baycrest, ON Canada; 5 Department of Neurology, Massachusetts General Hospital Harvard Medical School Boston, MA United States; 6 Norfolk and Norwich University Hospitals National Health Service Foundation Trust Norwich United Kingdom

**Keywords:** Alzheimer disease, dementia, spatial disorientation, getting lost, outdoor navigation, risk factors, environmental, GPS tracking, community, mobile phone

## Abstract

**Background:**

Spatial disorientation is one of the earliest and most distressing symptoms seen in patients with Alzheimer disease (AD) and can lead to them getting lost in the community. Although it is a prevalent problem worldwide and is associated with various negative consequences, very little is known about the extent to which outdoor navigation patterns of patients with AD explain why spatial disorientation occurs for them even in familiar surroundings.

**Objective:**

This study aims to understand the outdoor navigation patterns of patients with AD in different conditions (alone vs accompanied; disoriented vs not disoriented during the study) and investigate whether patients with AD experienced spatial disorientation when navigating through environments with a high outdoor landmark density and complex road network structure (road intersection density, intersection complexity, and orientation entropy).

**Methods:**

We investigated the outdoor navigation patterns of community-dwelling patients with AD (n=15) and age-matched healthy controls (n=18) over a 2-week period using GPS tracking and trajectory mining analytical techniques. Here, for the patients, the occurrence of any spatial disorientation behavior during this tracking period was recorded. We also used a spatial buffer methodology to capture the outdoor landmark density and features of the road network in the environments that the participants visited during the tracking period.

**Results:**

The patients with AD had outdoor navigation patterns similar to those of the controls when they were accompanied; however, when they were alone, they had significantly fewer outings per day (total outings: *P*<.001; day outings: *P*=.003; night outings: *P*<.001), lower time spent moving per outing (*P*=.001), lower total distance covered per outing (*P*=.009), lower walking distance per outing (*P*=.02), and lower mean distance from home per outing (*P*=.004). Our results did not identify any mobility risk factors for spatial disorientation. We also found that the environments visited by patients who experienced disorientation versus those who maintained their orientation during the tracking period did not significantly differ in outdoor landmark density (*P*=.60) or road network structure (road intersection density: *P*=.43; intersection complexity: *P*=.45; orientation entropy: *P*=.89).

**Conclusions:**

Our findings suggest that when alone, patients with AD restrict the spatial and temporal extent of their outdoor navigation in the community to successfully reduce their perceived risk of spatial disorientation. Implications of this work highlight the importance for future research to identify which of these individuals may be at an actual high risk for spatial disorientation as well as to explore the implementation of health care measures to help maintain a balance between patients’ right to safety and autonomy when making outings alone in the community.

## Introduction

### Background

Spatial disorientation is one of the earliest and most distressing symptoms seen in patients with Alzheimer disease (AD) [1,2]. It is defined as moments where patients are unsure about their whereabouts and are unable to navigate to an intended location [3]. This symptom manifests behaviorally as patients making navigation errors when in the community, which in turn can lead to a risk of them getting lost in both unfamiliar and familiar environments [4]. Being a prevalent problem worldwide, up to 70% of patients with dementia experience at least 1 getting lost episode over their disease course, while others experience multiple episodes [5-8]. Indeed, up to 40,000 people with dementia in the United Kingdom get lost in the community for the first time every year, and these incidence rates are likely to increase with the projected global rise in the patient population of dementia [5,9].

Although unpredictable in its onset, common real-world situations where patients with AD are likely to experience a getting lost episode include (1) when they perform routine activities in the community (ie, daily neighborhood walks and going to the corner shop), (2) when they are purposefully left unsupervised by their carer (ie, waiting for carer outside the shop), and (3) during night time while the carer is asleep [10,11]. Getting lost episodes can cause various negative consequences for the patients, such as increasing their chances of a care home admission by 7 times, decreasing their sense of autonomy, and increasing their risk of sustaining injuries and even potential death [7,12]. Extending beyond the patients themselves, other consequences of these episodes include increasing carer burden and distress as well as the involvement of law enforcement groups (ie, the police) and community search resources [11,13-15].

Despite getting lost episodes leading to significant negative consequences for the patients, their carers, and beyond, very little is still known about exactly why these episodes, and spatial disorientation in general, occur in patients with AD. From a neural standpoint, it has been suggested that spatial disorientation is seen more in AD as opposed to in other dementias [16,17]. Indeed, this is due to the pattern in which the AD neuropathology spreads, appearing early in regions of the brain that underlie spatial navigation. For example, neuropathology induced alterations to the medial temporal and parietal lobe structures result in impairments to egocentric (body-based) and allocentric (map-based) navigation strategies, respectively, as well as the interaction between the two [1]. Such navigation impairments can play a fundamental role in causing patients to make navigation errors when out in the community that they are ultimately unable to recover from, and hence leading to them getting lost.

In addition to the spatial navigation impairments, previous studies from our group have suggested that certain environmental factors, such as increased outdoor landmark density and complex road network structure, may act as risk factors for spatial disorientation by potentially triggering patients to make navigation errors [18,19]. However, these factors were identified using retrospective police case reports of missing people with dementia, and owing to the unavailability of trajectory data for the missing individuals, the true extent to which these factors contribute to spatial disorientation is unclear.

To date, very few studies have investigated the outdoor navigation patterns of patients with AD in the community, exploring these patterns in a general sense and, more specifically, relating them to factors such as caregiver burden and the individual’s own well-being [20-23]. However, none of these studies have related the measured navigation patterns of these individuals to the occurrence of spatial disorientation or environmental risk factors. Exploring this relationship can potentially offer insight into variables that are associated with spatial disorientation. Specifically, we are interested in mobility risk factors, which if identified can potentially be used to establish which individuals may be at a high risk for getting lost in the community.

### Aims

We thus conducted an outdoor navigation study on a sample of community-dwelling patients with AD and age-matched healthy controls, using GPS tracking over a 2-week period. Our first aim is to understand the outdoor navigation patterns of the patients over an extended period and in naturalistic, free-living conditions. Here, we wanted to investigate whether there are potential differences between healthy older adults and (1) patients overall, (2) patients when they are alone versus accompanied, and (3) patients who experienced and did not experience spatial disorientation in the tracking period. Our second aim is to test whether we could validate our previous study findings of environmental risk factors for getting lost episodes [18,19], by retrospectively investigating whether patients with AD experienced spatial disorientation when navigating through environments with a high outdoor landmark density and complex road network structure.

For our first aim, we present the following hypotheses (H):

First, Patients with AD would exhibit reduced outdoor navigation in the community when compared with healthy older adults based on findings from previous studies [20,22] and, more specifically, owing to the widely reported impairments in spatial navigation seen in patients with AD [1] (H1).

Second, we expect the potential reductions in outdoor navigation for the patients with AD to be relatively more apparent when they are alone than when they are accompanied on outings (H2).

Third, we also hypothesize that we will identify mobility patterns that support previously reported risk factors, which were identified through interviews and case reports, for spatial disorientation in patients. Specifically, patients who experience disorientation in the tracking period will have higher distances traveled from home (ie, venturing into unfamiliar environments) and have made increased nighttime outings into the community, thereby supporting commonly reported situations where spatial disorientation occurs for patients with AD using ecological outdoor navigation data [10] (H3).

For our second aim, we present the following hypothesis:

Patients who navigated through environments with both a high outdoor landmark density and complex road network structure will be the ones who experience spatial disorientation during the tracking period, as these 2 built features have been suggested as environmental risk factors for spatial disorientation in patients with AD by our previous studies [18,19] (H4).

## Methods

### Recruitment

A total of 16 community-dwelling patients with AD and 18 age-matched healthy controls were recruited to participate in this study (see Multimedia Appendix 1 for details). Before study participation, all participants underwent an initial telephone screening procedure to assess their eligibility for the study. The inclusion criteria were as follows: being aged between 50 and 80 years, living at home, and, if in the patient group, must have been given a clinical diagnosis for AD and have a carer (relative or spouse) that knows them well and who is willing to assist in the study. The exclusion criteria were having a previous history of alcohol or substance abuse, the presence of a psychiatric condition, the presence of any other significant medical condition that may be likely to affect participation in the study (head injury, loss of vision, and mobility issues), and for the patients, the presence of a comorbid neurological condition not related to AD.

Signed informed consent was obtained from all participants before undergoing the experimental protocol.

### Ethics Approval

Ethical approval for the study was provided by the Faculty of Medicine and Health Sciences Research Ethics Committee at the University of East Anglia (FMH2017/18-123) as well as the National Health Service Health Research Authority (project ID205788; 16/LO/1366).

### Experimental Protocol

All participants underwent an experimental protocol consisting of a cognitive testing session and 2-week GPS tracking (detailed in the next subsections).

#### Background Demographics and Cognitive Testing

The cognitive testing session for healthy controls was conducted in a quiet testing room at the university campus, and that for the patients, in a quiet room in their own home. Here, the background demographics of the participants including their age, sex, level of education, and whether they had any previous history of getting lost episodes were collected from their carers. In addition, the participants completed a range of cognitive tests and spatial navigation questionnaires. Of relevance to this study, the participants completed the Mini-Addenbrooke’s Cognitive Examination (Mini-ACE) and the Santa Barbara Sense of Direction (SBSOD) scale. The Mini-ACE is a sensitive, validated cognitive screening test for dementia, with lower scores indicating higher cognitive impairment; the SBSOD is a self-report scale that measures real-world environmental spatial abilities, with higher scores indicating higher spatial ability [24,25]. As the patients with AD may lack insight into their own navigational abilities because of the disease [26], we also asked their carers to complete the Spatial Orientation Screening (SOS) questionnaire. This is a newly developed screening tool that assesses the carer’s reports of the patient’s navigational impairments in the community, with higher scores indicating higher impairments [27].

#### GPS Tracking

After the cognitive testing session, all participants underwent GPS tracking of their outdoor navigation patterns in the community for a 2-week period, under naturalistic conditions. Here, outdoor navigation in the community is defined as any movement that occurs outside of the participant’s home and includes movement inside indoor locations in the community (eg, shopping malls and supermarkets). An exploratory time frame of 2 weeks was chosen for the tracking period to capture the participants’ outdoor navigation patterns over repeated weekdays and weekends as well as to account for potential day-to-day fluctuations in these patterns. Participants were tracked in parallel in groups of 3, with the entire data collection period spanning from November 2018 to November 2019 (ie, 12 months and 14 days).

All participants were visited at home and provided with a GPS tracker (Trackershop Pro Pod 5 [28]). They were instructed to wear the tracker (ie, by placing it in their coat or trouser pockets) whenever they left the house during the tracking period. All participants were asked to wear the tracker regardless of whether they were alone or accompanied and regardless of the mode of transport used when outside. For each outing, participants were asked to record the date and time of the outing, mode of transport used, and whether they were alone or accompanied during the outing in a navigation diary, which was provided to them as a template form. To account for the cognitive impairments seen in the patients, their carers were asked to ensure that they (ie, the patients) did not forget to wear the tracker whenever they left the house during the tracking period.

The GPS devices for the first batch of 22 participants (13 controls and 9 patients) recorded data of 1 sample every 3 seconds (ie, 0.33 Hz), whereas for the remaining 12 participants (5 controls and 7 patients), data were recorded of 1 sample every 5 seconds (ie, 0.20 Hz). The differences in sampling frequencies were due to the GPS company changing the lowest sampling frequency (from 0.33 to 0.20 Hz) of the devices on the web, midway through data collection. The devices recorded the following variables for each data point—date and time, address (street name), speed (kilometers per hour), battery level (percentage), distance traveled (kilometers), signal accuracy (percentage), and latitude and longitude coordinates.

#### Spatial Disorientation Behavior in the Tracking Period

Following the GPS data collection, we retrospectively obtained information about the spatial disorientation behavior of the patients during the tracking period from their carers. The carers were asked if there were any instances (that they knew of) in this period where their loved one experienced (1) a getting lost episode and (2) a subtler instance of spatial disorientation behavior, where the carers had to intervene and correct the navigation of the patient. On the basis of their carer’s responses, a simple yes or no for each disorientation behavior during the tracking period was recorded for all the patients.

### Data Analysis

#### GPS Trajectory Data Preprocessing

Preprocessing of the collected GPS trajectory data was carried out in MATLAB (version R2017b; MathWorks) and consisted of data cleaning, smoothing, and transportation mode classification.

For each participant, the data cleaning procedure involved identifying and removing days with no outdoor navigation from their data. Here, we identified 1 patient with almost no recorded data, owing to a faulty GPS tracker. This patient was removed for the analysis, leaving a total of 15 participants in the patient group. Following data cleaning, the data smoothing procedure was run on the remaining data of all participants, which involved identifying and removing spikes (ie, large high-frequency displacements in the data that reflect sensor noise or artifacts) in the data. Following recommendations in the literature, data points representing spikes were identified and removed using distance thresholds set between every consecutive pair of recorded data points (ie, the hypothetical distance that an individual could cover, assuming a set maximum speed, in the time difference between the data points) [29,30].

We next classified each participant’s trajectory data points into three transportation modes—stationary, by foot, and in vehicle. As a first step, we grouped all trajectory data points into time windows. For participants with data recorded at 0.33 Hz, each time window had a duration of 9 seconds, and for participants with data recorded at 0.20 Hz, each time window had a duration of 10 seconds. For both sets of participants, we set a duration for the time windows that was not only similar but also as small as possible, to ensure consistency between data recordings and to increase the accuracy of our transportation mode classification. Each time window was then classified into transportation modes (ie, *stationary*, *by foot*, and *in vehicle*) based on set mean and maximum speed values of the data points in that time window [31].

For further details of preprocessing (including distance thresholds for data smoothing and speed thresholds for transport mode classification), see Multimedia Appendix 1.

#### Outdoor Navigation Variables Analysis of GPS Trajectories

##### Overview

To explore the outdoor navigation patterns of the participants, total outings made, distance traveled (total and by foot), time spent moving outside, and distance traveled from home were all measured. These variables were selected as they have been suggested to represent important aspects of outdoor navigation in previous GPS tracking studies of people with dementia [20-22]. In addition, a study showed that the outings of people with dementia are dependent on time of day [20]. Thus, we also chose to look at total daytime and nighttime outings made to explore this pattern further. Finally, because qualitative findings from a previous study suggested that people with dementia stick to familiar routes when navigating in their neighborhood [32], the similarity of trajectories was our final variable of interest to investigate this pattern quantitatively.

##### Outings Made (Total, Daytime, and Nighttime)

From each participant’s trajectories, we identified the total number of outings they made. Here, an outing is defined as a journey that starts when the participant leaves their home and ends when they return home. Outings were identified by first calculating the distance of all recorded data points to the centroid of the participant’s home address. In line with previous research, all data points within 30 m (ie, 3 times the SD of the GPS device’s measurement error, allowing 97% confidence for determining true position) of the home address centroid (ie, GPS coordinates denoting the center of the private residence) were considered to reflect the participant being at home [33]. An outing was then identified whenever the participant’s trajectory left home and covered a minimum distance of 100 m, which has been shown to be a reasonable threshold to identify outings [34]. The total number of outings made by each participant over the tracking period was computed and normalized by dividing this value by the total number of recorded days.

Because of the influence of time of day on outdoor navigation in people with dementia [20], we were particularly interested in the total number of daytime (6 AM to 6 PM) and nighttime (6:01 PM to 5:59 AM) outings made. Although we recognize that the outdoor environments will have differing characteristics during these time bands according to the season (eg, amount of daylight), for consistency purposes, we used the same time bands for all participants, despite groups of participants being tracked at different times of the year. Keeping consistent time bands also has the advantage of accounting for variables apart from daylight alone that could influence participants leaving the house at different times of the day (eg, carer availability if typically working from 9 AM to 5 PM and rush hour pedestrian and vehicle traffic). The values of these variables were normalized for the total number of days that the GPS data was recorded.

##### Time Spent Moving Outside

For time spent moving outside home, the GPS devices used in this study automatically stopped recording data when no movement was detected for 2 minutes. Hence, for this variable, we calculated the sum of the total duration of each of the participant’s outings, excluding the periods where the participant was not moving. This variable was then normalized for the total number of outings made by the participant.

##### Distance Traveled (Total, by Foot, and From Home)

To compute total distance traveled, the distance between each pair of consecutive data points was summed across all the participant’s outings and normalized for the total outings made. The same method was used to calculate the distance traveled by foot, this time by using only the portions of each participant’s trajectories where they were walking (ie, walking trajectories). Again, this value was normalized for total outings made. To compute the distance traveled from home, we calculated the mean distance of the data points in each outing to the participant’s home and averaged this value across all outings.

##### Similarity of Trajectories

To compute our final variable of interest, similarity of trajectories, we used a metric known as the discrete Fréchet distance, which is derived from the continuous Fréchet distance metric [35]. The continuous Fréchet distance is used to assess the similarity of trajectories by measuring how similar 2 continuous curves are in their shape, considering the location and ordering of the data points that make up the curve [36]. A common example used to explain the concept of continuous Fréchet distances is that of a man walking his dog on a leash, where the man will be on one continuous trajectory (A) and the dog on another continuous trajectory (B). The continuous Fréchet distance refers to the minimum length of a line that is required to connect the man on trajectory A to the dog that is on trajectory B, with both walking forward simultaneously. The discrete Fréchet distance is a variation of this measure, whereby only the discrete data points that make up the trajectory (ie, the trajectory fixes) are considered, and all possible pairwise distances between the trajectories’ data points are assessed, with the maximum over all pairwise distances being the final computed value (see the study by Tao et al [37] for details). Here, the more similar the 2 trajectories are to each other, the lower the discrete Fréchet distance. We chose to use this metric as it works well for how our trajectory data set is structured, with the GPS data points for each participant being sampled at regular, discrete intervals. Furthermore, this metric gives a good approximation of the more comprehensive continuous Fréchet distance, is relatively inexpensive computationally, and has been used in previous studies for calculating trajectory similarity from naturalistic GPS data [37,38]. The discrete Fréchet distance DFD between 2 separate trajectories, *A* and *B*, is calculated using the formula as follows [35,37]:



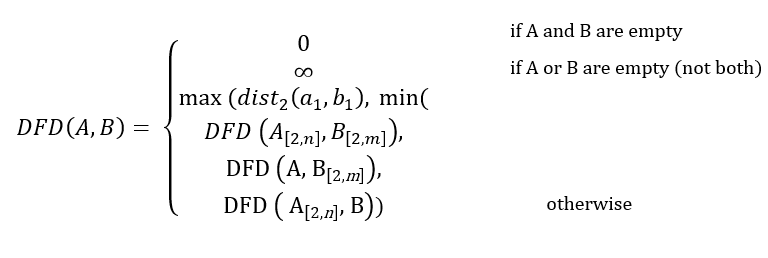



where *a*_1_ and *b*_1_ represent the first set of points in trajectories *A* of length *n* and *B* of length *m*. For each participant, we calculated the discrete Fréchet distances for all combinations of their outing trajectories using a MATLAB function [39], and computed the mean of these values.

**Figure 1:**
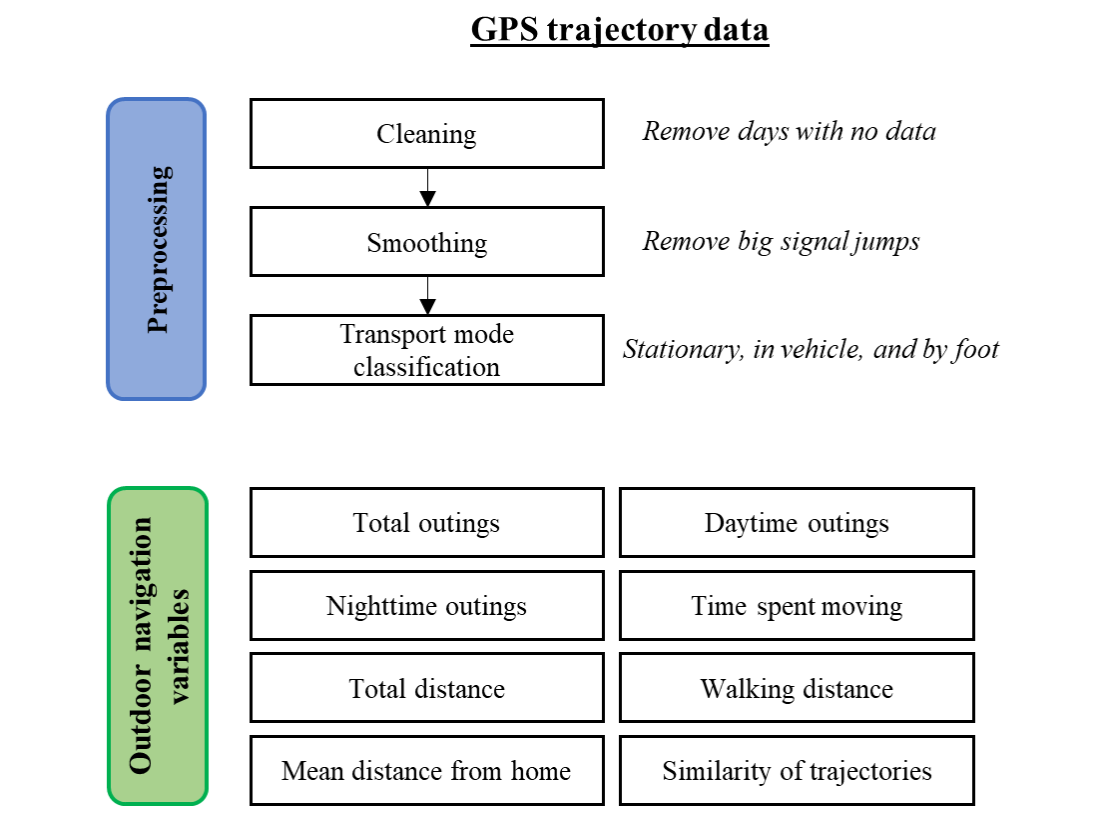
Overview of GPS trajectory data preprocessing procedure and summary of outdoor navigation variables used in this study. The collected GPS trajectory data from all participants undergo a data cleaning and smoothing procedure, followed by transport mode classification. In total, 8 outdoor navigation variables are then generated from the preprocessed data.

An overview of the GPS trajectory data preprocessing procedure and summary of all the outdoor navigation variables are illustrated in Figure 1.

##### Analysis Steps

We conducted our analysis in 3 different steps using RStudio software package (version 3.4.2) [40]. In the first step, we compared differences of all variables between the controls and patients using 2-tailed *t* tests. In the case of a nonnormal distribution, Wilcoxon rank-sum tests were used [41].

Then, in the second step, using information from the navigation diaries, we split the outings of each person with AD into outings made alone and outings made accompanied. The rationale for this is based on our prediction that the outdoor navigation patterns of the patients with AD would be influenced by whether they are alone or accompanied. When accompanied, they can rely on other individuals (ie, the carer) to navigate, whereas this is not possible when they are alone; hence, the latter situation is more likely to highlight patterns that are more reflective of their navigation impairments. For controls, we do not expect their outdoor navigation patterns to be influenced by whether they are alone or accompanied, owing to not having AD induced neuropathology that impairs navigational behavior, and hence did not split the data of this group. We then compared differences in all of the outdoor navigation variables across three groups—controls (all outings), patients (outings alone), and patients (outings accompanied). Linear mixed models were used to assess these differences using the *nlme* package in R [42], with group chosen as the fixed-effect, between-subjects factor and participant as the random-effect, within-subjects factor in the model. This statistical model was chosen as it accounts for participants in two of the groups (ie, patients when alone and patients when accompanied) being the same individuals, and the resulting interdependence that arises in the collected data of these individuals under both conditions. After running a separate mixed model for each variable, ANOVAs that were built in the R package were run to assess overall group significance, followed by post hoc pairwise tests (also built in the R package) that were corrected for multiple comparisons using the false discovery rate method [43].

For the final step, using the information on spatial disorientation during the tracking period that we obtained retrospectively from the carers of the patients, we divided these individuals into two groups (disoriented vs not disoriented during tracking period). We then investigated group differences in all the outdoor navigation variables across controls, patients with disorientation, and patients without disorientation using 1-way ANOVAs. In the case of a nonnormal distribution, Kruskal-Wallis tests were used [44].

**Figure 2:**
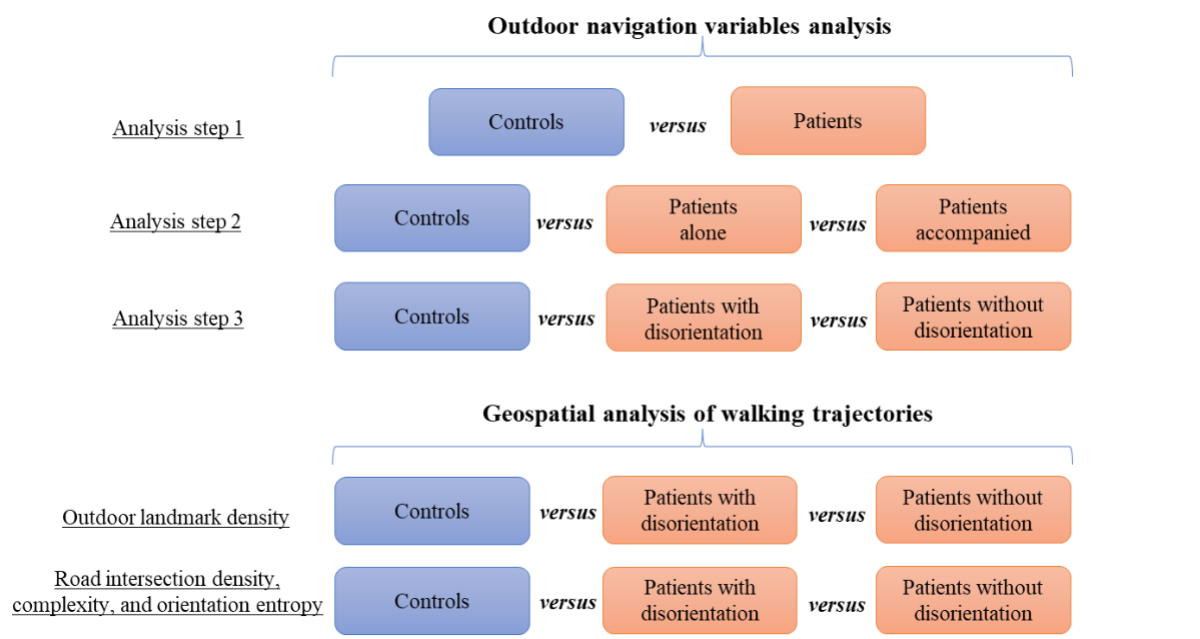
Overview of different analysis steps for the outdoor navigation variables analysis and geospatial analysis of walking trajectories. For the outdoor navigation variables analysis, a total of 3 between-group comparisons are made. For the geospatial analysis, between-group comparisons are made for the composition of the 2 different environmental variables (ie, outdoor landmark density and road network structure) in the buffer zones of the walking trajectories.

An illustration summarizing the different analysis steps for the outdoor navigation variables are provided in Figure 2.

##### Geospatial Analysis of GPS Trajectories

We conducted a geospatial analysis of our participants’ trajectories to test our findings that increased outdoor landmark density and complex road network structure may contribute to spatial disorientation in patients. For this, we imported and plotted each participant’s walking trajectories (ie, data points classified as by foot) into ArcGIS software (Esri) [45], using the World Geodetic System 1984 geographic coordinate system [46]. We chose to focus on only the participants’ walking trajectories as we assume that spatial disorientation is unlikely to occur for the patients when they are not walking (ie, passively sitting in vehicle). We recognize that disorientation can still occur for patients if they were actively driving a vehicle; however, we assume that none of the patients in our sample are active drivers given that they have cognitive impairments.

We first tested whether the patients who experienced disorientation during the tracking period showed walking trajectories that passed through areas with an increased outdoor landmark density. Here, we used the same outdoor landmark data set and spatial buffer methodology as in our previous study to measure the outdoor landmark density in the areas that all participants visited. In brief, the data set contained all outdoor landmarks that are visually accessible from open street view, and the methodology involved generating a spatial buffer zone around the trajectories to capture all landmarks surrounding the visited locations (see the study by Puthusseryppady et al [18] and Multimedia Appendix 1 for details). Here, we selected a radius of 50 m for the buffer zones generated around the participants’ walking trajectories, as previous studies have suggested this distance as being appropriate to capture all environmental features, such as outdoor landmarks, which are directly accessible along a traveled route [47,48]. To account for the measurement error in the GPS device (10 m), we added another 30 m to the buffer zones (ie, 3 times the SD of the measurement error to ensure 97% confidence for determining position) in addition to the initial 50 m, following guidelines in the literature [33]. Hence, for each participant, geodesic buffer zones of 80 m were generated around their walking trajectories, and the number of outdoor landmarks falling within these buffer zones (normalized for total walking distance) was then computed. Group comparisons on this variable were then made across the controls, patients with disorientation, and patients without disorientation using a Kruskal-Wallis test.

We next tested whether the patients who had experienced disorientation during the tracking period had walking trajectories that passed through areas with a high road intersection density and complexity. For this, we used the same road network data set and spatial buffer methodology as in a previous study (see the study by Puthusseryppady et al [19] and Multimedia Appendix 1 for details). In brief, the data set contained all roads and intersections in the United Kingdom, and the methodology involved generating a spatial buffer zone around the trajectories to capture all roads and intersections that were used by the participants on their outings. Here, to account for measurement error in the GPS device, a buffer zone radius of 30 m was chosen and generated around the participants’ walking trajectories. The number and average complexity of the road intersections (normalizing the former for total walking distance) falling within the buffer zones of all participants were computed, and group comparisons were made using Kruskal-Wallis and 1-way ANOVA tests, respectively.

Finally, we tested the impact of road orientation entropy in contributing to the patients experiencing spatial disorientation during the tracking period. Road orientation entropy measures the orientation of roads within a given area and is an indicator of how ordered the layout of the road network is within this area. Here, higher road orientation entropy indicates lower order, and lower road orientation entropy indicates higher order. As we found a buffer radius of 2 km to be sensitive to identify changes in road orientation entropy between different locations in our previous study [19], we chose to use this distance (plus a 30 m error buffer) for our buffer zones here. Subsequently, buffer zones of 2.03 km were generated around the participants’ trajectories, and the orientation entropy of the roads falling within these buffer zones were computed using the Shannon entropy (see the study by Puthusseryppady et al [19] and Multimedia Appendix 1 for details). Group comparisons were then made using a 1-way ANOVA.

An illustration summarizing the different analysis steps for the geospatial analysis of the GPS trajectories are provided in Figure 2.

## Results

### Participant Demographics

The controls and patients in this study did not differ statistically in their age or sex; however, a statistical difference was seen for number of years of education, with controls having higher number of years of education than the patients. The patients performed significantly worse than controls on the Mini-ACE; the scores of all these individuals met the upper cut-off of ≤25/30 for mild dementia and fall within ranges previously reported for patients with mild AD [25,49]. Most patients were reported by their carers to have a past history of at least 1 getting lost episode in the community (Table 1).

**Table 1:** Participant demographics.

		Controls (n=18)	Patients (n=15)	Significance, *P* value	
Age (years), mean (SD)		68.33 (7.53)	70.33 (6.86)	.40	
Education (years), mean (SD)		15.44 (3.11)	12.80 (1.78)	*.01* ^a^	
**Gender, n (%)**					.84
	Men	9 (50)	8 (53)		
	Women	9 (50)	7 (47)		
Mini-ACE^b^ score, mean (SD)		28.52 (1.50)	18.13 (5.64)	*<.001*	
Had getting lost history, n (%)		N/A^c^	12 (80)	N/A	

^a^Values in italics indicate a statistically significant group difference.

^b^Mini-ACE: Mini-Addenbrooke’s Cognitive Examination.

^c^N/A: not applicable.

### Outdoor Navigation Variables Analysis

The results of our first analysis of the outdoor navigation variables (controls vs patients) showed that overall, there were no significant group differences for any variable. However, when compared with those for the controls, trends were seen for patients making fewer nighttime outings (controls: mean 0.39, SD 0.32 outings; patients: mean 0.22, SD 0.24 outings; *P*=.09) and having a lower-distance traveled by foot (controls mean 1.95, SD 1.30 kilometers; patients mean 1.44, SD 1.10 kilometers; *P*=.07), but these results were not statistically significant.

The results of our second analysis (ie, after splitting the data of the patients into outings made alone and accompanied) showed significant group effects for 88% (7/8) of the variables (Table 2).

**Figure 3:**
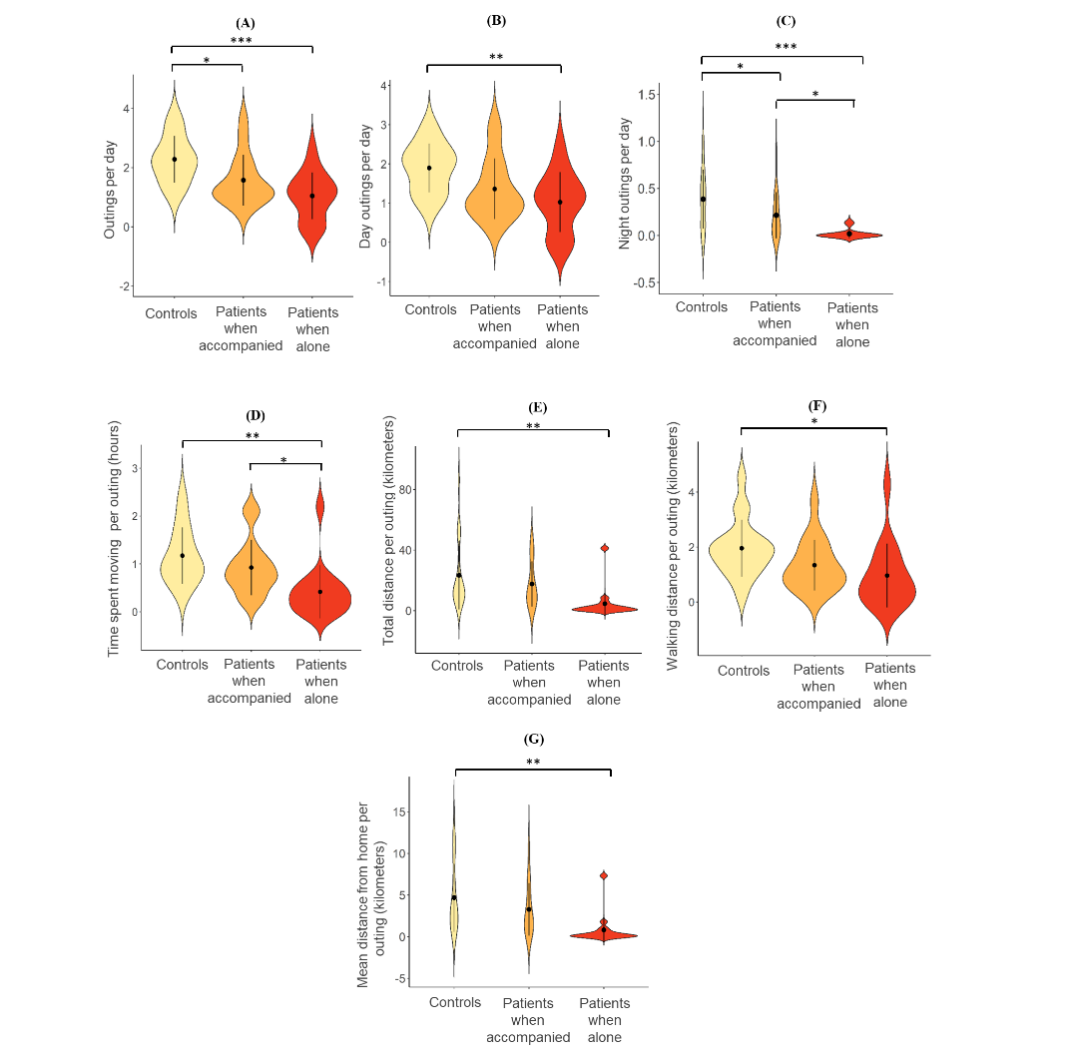
Violin plots of post hoc pairwise comparisons of the outdoor navigation variables. Single brackets show pairwise comparison, the waves represent a mirrored kernel density estimation of the probability distribution of the variables, the black dots indicate group means, and the lines intersecting the black dots indicate the group SDs: (A) outings per day, (B) day outings per day, (C) night outings per day, (D) time spent moving per outing, (E) total distance per outing, (F) walking distance per outing, and (G) mean distance from home per outing. Note that ranges of violin plots extend slightly above and below the actual range of data, as plots show smoothed-out distribution. *P<.05, **P<.01, ***P<.001.

Post hoc pairwise comparisons between the groups showed that compared with controls, patients when alone had significantly fewer outings per day (total outings—controls: mean 2.28, SD 0.79; patients alone: mean 1.04, SD 0.78; *P*<.001; day outings—controls: mean 1.89, SD 0.62; patients alone: mean 1.02, SD 0.76; *P*=.003; night outings—controls: mean 0.38, SD 0.31; patients alone: mean 0.01, SD 0.04; *P*<.001), lower time spent moving per outing (controls: mean 1.17, SD 0.58 hours; patients alone: mean 0.41, SD 0.55 hours; *P*=.001), lower total distance covered per outing (controls: mean 23.37, SD 22.64 kilometers; patients alone: mean 4.60, SD 10.40 kilometers; *P*=.009), lower walking distance per outing (controls: mean 1.94, SD 1.02 kilometers; patients alone: mean 0.94, SD 1.14 kilometers; *P*=.02), and lower mean distance from home per outing (controls: mean 4.69, SD 4.10 kilometers; patients alone: mean 0.80, SD 1.86 kilometers; *P*=.004; Figure 3). For the last variable (ie, similarity of trajectories across all outings), no significant differences were seen between these 2 groups. Meanwhile, when comparing the controls with patients when accompanied, no significant differences were seen in any of the variables except for total and night outings made per day. Here, compared with controls, patients when accompanied made significantly fewer total outings (controls: mean 2.28, SD 0.79; patients accompanied: mean 1.57, SD 0.85; *P*=.02) and night outings per day (controls: mean 0.38, SD 0.31; patients accompanied: mean 0.21, SD 0.24; *P*=.04; Figure 3). A trend was also seen for patients when accompanied making fewer day outings per day than the controls (controls: mean 1.89, SD 0.62; patients accompanied: mean 1.36, SD 0.77; *P*=.06); however, this result was not statistically significant. The above results are summarized in Figure 3.

When comparing patients when they were alone with when they were accompanied, significant differences were seen with patients when alone making fewer night outings per day and having less time spent moving per outing compared with when they were accompanied (night outings—patients accompanied: mean 0.21, SD 0.24; patients alone: mean 0.01, SD 0.04; *P*=.04; time spent moving per outing—patients accompanied: mean 0.92, SD 0.57 hours; patients alone: mean 0.41, SD 0.55 hours; *P*=.04; Figure 3). No significant differences were seen in any of the remaining variables, although compared with those for patients when they were accompanied, trends were seen for patients when alone having fewer total outings per day (patients accompanied: mean 1.57, SD 0.85; patients alone: mean 1.04, SD 0.78; *P*=.09), lower total distance per outing (patients accompanied: mean 17.63, SD 14.90 kilometers; patients alone: mean 4.60, SD 10.40 kilometers; *P*=.08), and lower mean distance from home per outing (patients accompanied: mean 3.28, SD 3.15 kilometers; patients alone: mean 0.80, SD 1.86 kilometers; *P*=.07), however these results were not statistically significant.

To explore whether interindividual differences in the outdoor navigation variables for the patients when alone were related to their subjective perception of spatial ability, we correlated their output on all variables (on outings alone) with their respective scores on the SBSOD scale, as a post hoc analysis. We also explored whether their output on the outdoor navigation variables on outings alone were related to their navigation impairments as reported by their carers, by correlating these variables with their scores on the SOS as well. Pearson correlations and Spearman correlations (for nonnormally distributed variables) were run for this. The results showed no significant correlations between patient scores on either the SBSOD or the SOS with any of the outdoor navigation variables.

For our third analysis, we found that none of the patients were reported by their carers to have gotten lost during the tracking period. However, 6 individuals were reported to have experienced more subtle moments of spatial disorientation, where they did not get lost but their carer had to intervene and correct their navigation. The results did not show any significant group differences for any of the outdoor navigation variables (Table 3).

**Table 2:** Comparison of outdoor navigation variables (controls vs patients accompanied vs patients alone).

Outdoor navigation variable	Controls, mean (SD)	Patients accompanied, mean (SD)	Patients alone, mean (SD)	Group significance, *P* value	Post hoc (controls—patients accompanied), *P* value	Post hoc (controls—patients alone), *P* value
Outings per day	2.28 (0.79)	1.57 (0.85)	1.04 (0.78)	*<.001* ^a^	*.02*	*<.001*
Day outings per day	1.89 (0.62)	1.36 (0.77)	1.02 (0.76)	*.004*	.058	*.003*
Night outings per day	0.38 (0.31)	0.21 (0.24)	0.01 (0.04)	*<.001*	*.04*	*<.001*
Time spent moving per outing (hours)	1.17 (0.58)	0.92 (0.57)	0.41 (0.55)	*.001*	.22	*.001*
Total distance per outing (kilometers)	23.37 (22.64)	17.63 (14.90)	4.60 (10.40)	*.011*	.34	*.009*
Walking distance per outing (kilometers)	1.94 (1.02)	1.33 (0.91)	0.94 (1.14)	*.04*	.14	*.02*
Mean distance from home per outing (kilometers)	4.69 (4.10)	3.28 (3.15)	0.80 (1.86)	*.005*	.21	*.004*
Similarity of trajectories across outings (mean discrete Fréchet distances)	0.14 (0.13)	0.09 (0.08)	0.04 (0.09)	.10	.30	.12

^a^Values in italics indicate a statistically significant group difference.

**Table 3:** Comparison of outdoor navigation variables (controls vs patients with disorientation vs patients without disorientation).

Outdoor navigation variable	Controls, mean (SD)	Patients with disorientation, mean (SD)	Patients without disorientation, mean (SD)	Group significance, *P* value
Outings per day	2.28 (0.79)	1.70 (0.71)	2.11 (0.92)	.25
Day outings per day	1.89 (0.62)	1.49 (0.62)	1.87 (0.80)	.79
Night outings per day	0.38 (0.31)	0.20 (0.19)	0.23 (0.27)	.24
Time spent moving per outing (hours)	1.17 (0.58)	1.13 (0.75)	0.82 (0.44)	.17
Total distance per outing (kilometers)	23.37 (22.65)	21.62 (16.41)	15.47 (13.85)	.60
Walking distance per outing (kilometers)	1.94 (1.02)	1.94 (1.49)	1.09 (0.61)	.06
Mean distance from home per outing (kilometers)	4.69 (4.10)	4.13 (3.13)	2.81 (2.89)	.34
Similarity of trajectories across outings (mean discrete Fréchet distances)	0.14 (0.13)	0.11 (0.09)	0.09 (0.08)	.59

### Geospatial Analysis of GPS Trajectories

Our first set of results for the geospatial analysis showed that there was a significant group difference in the outdoor landmark density surrounding the walking trajectories (*P*<.001). Post hoc pairwise Wilcoxon rank-sum tests showed that the walking trajectory buffer zones of the controls had a significantly higher outdoor landmark density than that of the patients with and without disorientation (*P*=.002 and *P*<.001, respectively). However, there were no significant differences when comparing the outdoor landmark density falling within the walking trajectory buffer zones of the patients with disorientation with those without (*P*=.60).

Our second set of results showed that there were no significant group differences in the density or complexity of the road intersections that were encountered by the participants’ walking trajectories (*P*=.43 and *P*=.45, respectively). Our final set of results showed that there was a significant group difference in the road orientation entropy surrounding the participants’ walking trajectories (*P*=.01). Post hoc pairwise *t* tests showed that the road orientation entropy surrounding the walking trajectories of controls was significantly higher than that of the patients with and without disorientation, respectively (*P*=.03 for both). However, there were no significant differences seen in the road orientation entropy surrounding the walking trajectories of the patients with disorientation and those without (*P*=.89).

## Discussion

### Principal Findings

We found that patients with AD overall did not exhibit any significant differences in their outdoor navigation in the community when compared with the controls, which was not in support of our hypothesis H1. However, after dividing the patients’ data into outings made alone and accompanied, we found that when alone, patients exhibited lesser and more restricted outdoor navigation in the community compared with the controls, which supports our hypothesis H2. When they were accompanied, most of their outdoor navigation patterns were similar to those of the controls; they only differed from controls in terms of their number of daytime and nighttime outings. Furthermore, our results did not highlight any significant mobility risk factors for spatial disorientation in the patients with AD, which was not in support of hypothesis H3, and finally, we did not find an association between increased outdoor landmark density and complex road network structure with spatial disorientation in these individuals, which was not in support of hypothesis H4.

In more detail, our results showed that on outings alone, patients cover lower distances (total and walking), spend less time moving outside, and stay closer to home, with the latter 2 findings being in line with previous studies [20,22]. Expanding on the finding from one of these studies that the timing of outings made by patients with AD are less varied than that by controls [20], we show here that patients make less daytime and nighttime outings when alone. Furthermore, it has previously been reported qualitatively that patients with AD stick to using familiar routes in their neighborhood [32]. Our findings did not corroborate these previous findings, as we found no significant differences in the similarity of routes taken by controls and patients, regardless of whether the latter were on outings alone or accompanied. However, it is worth mentioning here that measures of route similarity are likely to be influenced by differences in environmental constraints seen across the locations that the participants have navigated (ie, having few vs various route options), which we did not consider here. Further, the discrete Fréchet distance metric used here is one of many that can be used to compute trajectory similarity. Whether our results still hold true when considering other trajectory similarity measures, such as dynamic time warping and longest common subsequence [37], remains to be investigated by future studies. Overall, this is the first study, to the best of our knowledge, that has systematically investigated differences in the outdoor navigation patterns of patients with AD in the community when they were alone versus accompanied.

It is apparent that the restricted outdoor navigation patterns seen in patients with AD on outings made alone is associated with spatial disorientation, with the carers of most of these individuals (n=11) indicating on the SOS questionnaire that the patients refrain from traveling and participating in activities alone owing to them (ie, the patient) being worried about finding their way. With most of the patients in our sample having had a previous history of getting lost in the community, our findings reflect a method adopted by these individuals (likely in response to these episodes) to reduce the risk of them experiencing spatial disorientation. Indeed, this risk reduction strategy agrees with a previous study that reported that restricting outdoor navigation to very familiar locations acts as a protector against getting lost for patients with AD [50]. However, further to a fear of spatial disorientation, other factors may also explain the restricted navigation patterns of patients when alone, including physical mobility and visual acuity impairments, as well as fear of accidents and falling and so on, which were not measured here. In addition to the patients themselves, we also consider the potential influence that their carers may have on the adoption of this risk reduction strategy, particularly regarding them being hesitant toward the patient making outings alone. Therefore, it is likely that the combination of external intervening behavior from the carers and the internal curtailing of navigation behavior by the patients themselves underlie their restricted outdoor navigation patterns when alone. To the best of our knowledge, this is the first study to relate the outdoor navigation patterns of patients with AD in the community to spatial disorientation, with previous studies having only related these patterns to caregiving burden and the individual’s own well-being [21,23].

We were unable to identify significant mobility risk factors for spatial disorientation in the patients with AD, suggesting that spatial disorientation cannot be explained by looking solely at how these individuals move in the community. However, considering that patients restrict their outdoor navigation to reduce their risk of spatial disorientation, it could very well be that the variables that they are restricting actually reflect risk factors for spatial disorientation. Along these lines, increased daytime and nighttime outings, time spent moving outdoors, distance traveled (total and walking), and traveling further away from home may all represent factors that increase the likelihood of patients experiencing spatial disorientation. Further research is required to determine whether any of these variables truly represent mobility risk factors for spatial disorientation in the community. Another point worth mentioning is that although we analyzed the spatial and temporal extent of the participants’ outings, we did not record information on the purposes of the outings made. It may be that contextualizing the patients’ outings offer further insight into potential mobility risk factors for spatial disorientation (ie, patients may experience disorientation when making a certain kind of outing), which is indeed a factor worth exploring in future studies. Our geospatial analysis of the GPS trajectories showed that the areas visited by patients who did and did not experience disorientation had a similar outdoor landmark density and complexity of road network structure. This null result suggests that we are not able to validate our findings from our previous studies at this stage [18,19]. This discrepancy in results could likely be due to differences in sample size, with this study having only 6 patients with spatial disorientation compared with the much larger sample of 210 individuals in our previous studies. Moreover, there was a lack of clarity on the specific locations where the patients felt disoriented in this study because disorientation was measured retrospectively by carer responses after the outings happened, whereas the previous studies used a spatial buffer analysis on locations from where patients with dementia were reported to have experienced spatial disorientation and went missing from. It is also possible that, owing to the carer having a personal relationship with the patient, their noting of patient disorientation may have been influenced by their previous navigational experiences with the patient (ie, falsely identifying disorientation in moments where patients may not actually be disoriented). To overcome these limitations, future studies should attempt to replicate our investigation using a relatively larger sample size of disoriented patients with AD, as well as a finer grained buffer analysis on the specific locations where this behavior occurred. Future studies may also look to use sensor-based measurements of navigation activity, which may be more accurately able to infer participant disorientation in specific environments using machine learning approaches that can identify whether participants exhibit deviations from performance benchmarks.

Although the risk reduction strategy of restricting outdoor navigation suggests that patients are aware of their navigation impairments when in the community, our post hoc analysis results showed no correlations between patient scores on the SBSOD scale and their outdoor navigation behavior when alone. Although the exact reason for this is unclear at present, with SBSOD scale scores having shown to correlate with scores on specific navigation tasks (learning new spatial layouts, making directional judgments in familiar environments, etc) [24], the lack of explicit measures of navigation ability in our outdoor navigation variables could explain this null result. We also did not find any relationship between patient scores on the SOS questionnaires and their outdoor navigation behavior when alone. This null result could be due to the SOS questionnaire being a new and yet to be validated instrument [27]; hence, the extent to which it relates to ecological measures of outdoor navigation in the community is unclear. More importantly, it can be argued that the carers’ responses on the second half of the SOS questionnaire (ie, using a Likert scale to rate the patient’s current navigation abilities compared with how it was in the past) can potentially be influenced by their own anxiety levels about the condition of the patient. As these responses can potentially factor into the overall questionnaire score, it may very well be that these scores may not be reflecting the true extent of patients’ navigation impairments. Finally, the relatively low variability between patients in their outdoor navigation variables when alone could also be a factor underlying the null correlations seen with their scores on the SBSOD scale and SOS questionnaire.

The finding of patients exhibiting significantly less outings, distance traveled, and time spent moving outside when they were alone has significant implications in how health care professionals can help manage well-being and independence in these individuals. Given the importance of outings for cognitive and physical health [51], as well as quality of life and psychosocial well-being [52], health care practitioners should advise that, at least in instances where there is a previous history of getting lost, patients with AD are accompanied regularly for outings. Indeed, this activity can potentially help maintain the ability to perform daily functions for the patients, thereby reducing their risk of institutionalization and alleviating caregiver burden in the long term [53]. However, considering that this may place an increased burden on the carer, the implementation of future technologies that can enable patients with AD to feel more at ease and assist their navigation when making independent outings should be explored. This can potentially include investigating the effect of wearing GPS trackers in evoking feelings of safety when going on outings alone, as well as the use of augmented reality, which may be able to use street maps to assist patients with AD with provided directions to their home when on outings alone.

### Limitations

Despite our novel findings, there are some limitations to our study that need to be addressed. We did not consider the extent to which premorbid lifestyle patterns may explain the restricted outdoor navigation patterns seen in the patients on outings alone. Because of our limited sample size, we also did not investigate further the effect of gender and different age groups, both of which have been suggested as factors influencing outdoor navigation patterns [20,54]. Future studies should focus on patients who have not yet gotten lost before and investigate longitudinally the effect that the incidence of a getting lost episode has on changes in their outdoor navigation patterns, including how this varies by gender and age. This approach would not only help gain a more holistic view of how outdoor navigation patterns are affected in patients owing to spatial disorientation but also potentially help identify mobility risk factors for spatial disorientation and getting lost episodes in these individuals as well. In addition, we also did not consider interindividual differences in use of technology during navigation, which could have influenced the results as it is possible that patients who are more competent with navigation aid devices such as smartphones may be less likely to experience spatial disorientation during their outdoor navigation. Future studies investigating spatial disorientation over an extended period could control for this potential confound by recruiting patients with minimal everyday use of navigation aid devices, to ensure accurate capturing and reporting of spatial disorientation episodes.

### Conclusions

In conclusion, our results showed that patients with AD when alone restrict the spatial and temporal extent of their outdoor navigation in the community to reduce their risk for experiencing spatial disorientation. From a research perspective, our findings highlight the potential for exploring navigation patterns before getting lost episodes occur to identify mobility risk factors that may contribute to spatial disorientation. Furthermore, our results underscore the utility of using GPS tracking to elucidate the causal impact of environmental variables on spatial disorientation. Our findings also have ethical implications. Restricting outdoor navigation in the community can have a negative impact on the patients’ autonomy and overall quality of life [55]. Hence, this may not be the most appropriate solution to the problem as not all these individuals may actually be at a high risk for experiencing spatial disorientation in the community. To strike a balance between their right to autonomy and safety, an important step for future studies is to identify which patients are indeed at a high risk for spatial disorientation by assessing their navigation performance in naturalistic community settings. Identifying such a group would in turn have clinical implications, as more measures can be implemented into the safeguarding plan of these individuals to prevent them from getting lost in the community in the future.

## Appendix

Multimedia Appendix 1 Methods of participant recruitment, data preprocessing, and geospatial analysis over and above what is already included in the main text.

## Abbreviations

AD: Alzheimer disease
Mini-ACE: Mini-Addenbrooke’s Cognitive Examination
SBSOD: Santa Barbara Sense of Direction
SOS: Spatial Orientation Screening
