# Supplementary material for: Using GPS Tracking to Investigate Outdoor Navigation Patterns in Patients With Alzheimer Disease: Cross-sectional Study
Source: JMIR Aging. 2022 Apr 21;5(2):e28222. doi: 10.2196/28222 (PMC9073623; doi:10.2196/28222)
Supplement: Multimedia Appendix 1 [file aging_v5i2e28222_app1.docx]

# Supplementary Information

## Participant Recruitment

The patients were recruited from three main sources. The majority of patients were recruited from a research clinic run by our team; patients are in the first instance referred to this clinic from clinicians to participate in dementia research. Some patients were also recruited from the Join Dementia Research website (<https://www.joindementiaresearch.nihr.ac.uk/>), which is an online service allowing individuals with memory problems/dementia, carers of such individuals, and healthy individuals to self-register to participate in dementia research studies. Lastly, some patients were also recruited from memory and dementia cafes/fayres held by our study team.

Recruitment of the healthy controls included individuals who had attended the research clinic as well as individuals who had participated in other studies conducted by our team and who had also given consent to be contacted for future research. In addition, individuals who expressed their interest in the study as a result of word of mouth were also recruited as controls for the study.

## GPS Trajectory Data Pre-Processing

### Data Smoothing

The data smoothing procedure involved identifying and removing large displacements reflecting sensor noise/artefacts between the data points. These displacements represent random errors in the data collection process, and occur due to various reasons including issues with the satellite or receiver, troublesome weather or atmospheric disturbances, and the urban canyon effect (i.e., GPS signal being reflected by tall buildings and surfaces). One of the most straightforward methods that has been suggested in the literature to identify such displacements is setting and using distance thresholds between data points. A distance threshold refers to the maximum distance that an individual could hypothetically cover in the time difference between two successively recorded data points, assuming that they are travelling at a certain speed. Since the GPS trajectories of all participants contains a mixture of transportation modes (i.e., they have not yet been classified according to transport modes), the maximum speed that our participants can travel, regardless of their transport mode, is 70mph; considering this maximum speed value and the GPS device’s sampling frequencies of 0.33 Hz/0.20 Hz, we can assume that the distance between any two data points should not realistically exceed a threshold of 93.87/156.45 metres respectively. After setting these thresholds, for each participant we identified and removed data points which had distances to the point immediately before and immediately after that exceeded these thresholds (i.e., displacements).

### Transport Mode Classification

The GPS trajectory data points of each participant were classified into three main transport modes – stationary, by foot, and in vehicle. As a first step, we grouped all trajectory data points into time windows. For participants with data recorded at 0.33 Hz, every 3 data points were grouped into a single time window; hence a single time window had a duration of 9 seconds. Meanwhile, for participants with data recorded at 0.20 Hz, every 2 data points were grouped into a single time window; hence for these participants a single time window had a duration of 10 seconds. Then, each time window was classified into one of the three transport modes based on the mean and maximum speed values of the data points in that time window. A time window is classified as:

- ‘Stationary’ if the mean speed was 0mph
- ‘By foot’ if the mean speed was greater than 0mph but less than or equal to 4mph and had a maximum speed value of less than or equal to 4mph.
- ‘In vehicle’ if the mean speed was greater than 4mph or if the mean speed was less than or equal to 4mph and had a maximum speed of greater than 4mph.

After the initial classification of all time windows into the different transport modes, a false positive check was conducted to refine this process. Regarding time windows classified as ‘By foot’, if such time windows were immediately preceded and proceeded by time windows classified as ‘In vehicle’ (eg. In vehicle, By foot, In vehicle), we can assume that these ‘By foot’ time windows are false positives. This is because such cases would realistically suggest instances when the vehicle (that the participants were in) was travelling really slowly, as opposed to suggesting instances where the participant got out of the vehicle, walked for 9-10 seconds, and got back in the vehicle. Here, these false positives were relabelled to ‘In vehicle’. On the flipside, if time windows classified as ‘In vehicle’ were immediately preceded and proceeded by time windows classified as ‘By foot’ (eg. By foot, In vehicle, By foot), then these were also identified as false positives. This is because it is not realistic for a participant to have a 9-10 second period of being in a vehicle in the middle of a period of walking. Here, these false positives were relabelled to ‘By foot’. After relabelling all false positives, all time windows labelled ‘In vehicle’ and ‘Stationary’ were filtered out, leaving only portions of each participant’s trajectory data where they were on foot (i.e., walking trajectories).

### Walking Trajectories – Data Smoothing

Before calculating the distance travelled by foot, we first identified and removed large displacements in each participant’s walking trajectory dataset by once again using the distance threshold method as used for the overall dataset. Here, considering a maximum speed of 4mph, the distance thresholds between any two data points were set to 5.36 metres and 8.94 metres for participants with data recorded at 0.33 Hz and 0.20 Hz respectively. All data points in the walking trajectories which exceeded these thresholds (i.e., displacements) were then identified and removed.

## Geospatial Analysis of GPS Trajectories

To run the geospatial analyses on the participants’ GPS trajectories, an outdoor landmark and road network datasets were used.

### Outdoor Landmark Dataset

A dataset containing all landmarks for the UK, in shape-file format, was downloaded from OpenStreetMap and imported into ArcGIS. The landmarks in this dataset represented any object or location that belonged to one of the following five categories – Amenity & Leisure, Tourism, Traffic & Transport, Urban & Rural Furniture, and Historic. For each landmark, the dataset contained details of its name (e.g., Riverside Leisure Centre), type (Swimming Pool), and map co-ordinates (X,Y; in the World Geodetic System 1984 geographic co-ordinate system). The landmarks in this dataset were overlaid onto a map of the UK; both maps were using the World Geodetic System 1984 geographic co-ordinate system.

For pre-processing the landmarks, we first identified and removed duplicate landmarks in the dataset. After this, landmarks falling inside other landmarks were identified and their visibility from open street view was assessed using Google Maps. Here, landmarks not visible from open street view (e.g. individual shops falling inside a shopping mall) were removed from the dataset, with the rationale that it would be unlikely for the participants to have been exposed to or used these landmarks during their outdoor navigation. On the other hand, landmarks falling inside other landmarks that were visible from open street view were examined to assess if they were at least as salient as the landmark they fell within, using Google Maps. Provided that this condition was met, (e.g., a bell tower that is part of a church courtyard), these landmarks were kept in the dataset, with the rationale that it may have been equally as likely for either of these landmarks to have caught the attention of/been used by the participants during their outdoor navigation. If this saliency condition was not met, then those landmarks were removed from the dataset. The landmarks that were remaining in the dataset, after all the pre-processing steps, were used for the spatial buffer analysis.

### Road Network Dataset

A dataset containing all the roads (major & minor) and intersections in the UK were downloaded from the Ordnance Survey Open Roads layer (https://www.ordnancesurvey.co.uk/business-government/products/open-map-roads). Here, road intersections were represented as vertices and the roads were represented as edges connecting the vertices. All roads and intersections for the UK were overlaid onto a map of the UK, for the spatial buffer analysis.

To calculate road orientation entropy, we first calculated the angular orientation of each road (i.e., angle between compass North and start/end points of road) falling within the participants’ trajectory buffer zones. The subsequent orientation values of all roads in the patients’ and controls’ trajectory buffer zones were grouped into 36 bins, with each bin representing incremental ranges of 10⁰ (i.e., 0-10, 11-20, 21-30…351-360). Shannon’s entropy (H) was then computed for the distribution of road orientations across all bins for the patients’ and controls’ trajectory buffer zones, using the formula:

H $= -\sum_{i=1}^{n} P\left( 0_{i} \right) \log_{e} P(0_{i})$

Where *n* is the total number of bins, *i* is the bin number, and P(0*_i_*) is the probability of a randomly selected road from the sample falling in bin number *i*.
